# Evaluation of the Mawid mobile healthcare application in delivering services during the COVID-19 pandemic in Saudi Arabia

**DOI:** 10.1093/inthealth/ihab018

**Published:** 2021-04-16

**Authors:** Turki M Alanzi, Arwa Althumairi, Afnan Aljaffary, Asma Alfayez, Demah Alsalman, Fahad Alanezi, Hala Alhodaib, Maha M AlShammari, Reem AL-Dossary, Saja Al-Rayes, Beyan Hariri, Bashair AlThani

**Affiliations:** Health Information Management and Technology Department, College of Public Health, Imam Abdulrahman Bin Faisal University, Saudi Arabia; Health Information Management and Technology Department, College of Public Health, Imam Abdulrahman Bin Faisal University, Saudi Arabia; Health Information Management and Technology Department, College of Public Health, Imam Abdulrahman Bin Faisal University, Saudi Arabia; Health Information Management and Technology Department, College of Public Health, Imam Abdulrahman Bin Faisal University, Saudi Arabia; Health Information Management and Technology Department, College of Public Health, Imam Abdulrahman Bin Faisal University, Saudi Arabia; Community College, Imam Abdulrahman Bin Faisal University, Damamm, Saudi Arabia; Department of Community Health Sciences, College of Applied Medical Sciences, King Saud University, Riyadh, Saudi Arabia; Computational Unit, Department of Environmental Health, Institute for Research and Medical Consultations, Imam Abdulrahman Bin Faisal University, Dammam, Saudi Arabia; College of Nursing, Imam Abdulrahman Bin Faisal University, Dammam, Saudi Arabia; Health Information Management and Technology Department, College of Public Health, Imam Abdulrahman Bin Faisal University, Saudi Arabia; Health Information Management and Technology Department, College of Public Health, Imam Abdulrahman Bin Faisal University, Saudi Arabia; College of Business Administration, Imam Abdulrahman Bin Faisal University, Dammam, Saudi Arabia

**Keywords:** COVID-19, ease of use, Mawid, mobile application, satisfaction

## Abstract

**Purpose:**

The purpose of this study is to evaluate MAWID mobile application developed by the Ministry of Health, Saudi Arabia, which is used for primary care hospitals appointments management and for tracking and tracing COVID-19.

**Participants and Methods:**

An online questionnaire-based survey was used for collecting data related to three major factors including Ease of Use, Satisfaction, and Benefits of MAWID application among its users. Out of total 2542 participants, 345 participants completed only a part of the survey, and 204 participants did not use the application. After removing, 549 invalid responses, a final sample of 1993 was included for the data analysis.

**Results:**

82.1% of the participants referred MAWID as easy to use application, 79.8% were highly satisfied with the application, and majority of the participants reflected potential benefits of using the application. T-test results have revealed that significant differences existed between males and females, and young and older participants in relation to the Ease of Use and Satisfaction levels associated with MAWID application.

**Conclusion:**

Mobile applications can be very effective in delivering the healthcare services during pandemics. However, there is a need for regular evaluation and assessment to trach the change in users′ needs and update the app according to the changing requirements.

## Introduction

The frequency in the occurrence of epidemics has been increasing in the past few years. Various epidemics, including severe acute respiratory syndrome coronavirus (SARS-CoV), Middle East respiratory syndrome, Ebola, Zika virus, have been identified in the past few years^1^ in different regions. In addition, the recent pandemic, coronavirus disease 2019 (COVID-19), has affected all aspects of life—social, economic, cultural and political. Lifestyles and access to services have been drastically affected, which may continue for some time. The total number of COVID-19 cases has passed 10 million, including >5 million deaths worldwide as of 1 July 2020.^2^ Two groups of people were identified to be at higher risk due to COVID-19: older people (>60 y of age) and those with underlying medical conditions (such as cardiovascular disease, diabetes, chronic respiratory disease and cancer).^3^ These two groups are considered to be those who require regular access to healthcare services such as diagnosis, assessment and treatment. However, with the rapid increase in the number of COVID-19 cases, most of the hospital resources were allocated to the treatment of COVID-19 patients, which has significantly affected patients with non-communicable diseases. It is estimated that >53% of countries have partial or complete disruption in the delivery of services to patients with hypertension, 49% for diabetic patients, 42% for cancer patients and 31% for cardiovascular patients.^4^ In addition, 63% of countries identified disruptions in rehabilitation services and 93% of countries partially or fully reassigned healthcare staff to treat COVID-19 patients.^4^ Disruptions in such services have significantly affected large numbers of patients who might need healthcare services such as dialysis, pregnancy monitoring and counselling, therapies and diagnosis. For example, doctors in the USA switched to less-frequent in-person appointments as concerns regarding the contamination of hospitals with SARS-CoV-2 have increased.^5^ In relation to the care of pregnant women, the Centers for Disease Control and Prevention in the USA stated that some doctors might find it necessary to separate the woman and child immediately after birth if they suspect that the woman may have contracted SARS-CoV-2, while in the UK, hospitals have banned all in-person visits to the wards.^6^^,^^7^ Therefore both access to services and changes in healthcare settings and procedures for service delivery have been noticed due to the COVID-19 pandemic.

In addition, COVID-19 also affected the healthcare economy, as significant reductions in primary healthcare services (up to 70%) have been identified and the salaries of healthcare staff have been either reduced or frozen, resulting in issues affecting both healthcare providers and patients.^8^ The healthcare system in Italy was on the verge of collapse with the rapid increase in COVID-19 cases from March to May 2020. Intensive care units were full and many patients were put on hold or had to be rejected due to the unavailability of hospital beds.^9^ Further increasing the complexity in healthcare services delivery, >1500 healthcare staff became ill, with 14% of confirmed COVID-19 cases among the doctors and other staff in Spain.^10^ With the increasing burden on the healthcare system, the situation may get even worse once the pandemic is over, adding strain on an already taxed workforce.^11^ Therefore there is a need for preparedness and planning for managing healthcare services during and in the aftermath of COVID-19.

With increasing healthcare challenges various countries, including BRICS nations (Brazil, Russia, India, China and South Africa), G7 nations (Canada, France, Germany, Italy, Japan, the UK and the USA) and low- and middle-income countries, have significantly increased medical spending with a focus on increasing healthcare efficiency and promoting remote healthcare technologies.^12^^–^^14^ Russia is one of the first countries in the world to implement preventive and sanitary measures, with 100 000 professional staff fully employed in this service. Sanitary quarantine centres have been established across all the major public spaces, including airports, railway stations and pedestrian crossings, to prevent the spread of COVID-19.^15^

The situation is not very different in Saudi Arabia. Since the start of the pandemic there have been 190 823 confirmed cases, including 1649 deaths.^16^ The country has taken various preventive measures that were applauded by many, such as cancelling the Hajj pilgrimage, implementing curfews and restricting travel.^17^ As a result, the increase in the number of new cases is slow compared with the rest of the world. In addition, providing free treatment for COVID-19 patients,^18^ increasing private-sector participation in managing COVID-19 regulatory process^19^ and international pacts on healthcare management^20^ are a few of the major initiatives taken to improve the preparedness of the healthcare system during and after COVID-19. One of the most reliable strategies being implemented for managing healthcare services includes healthcare delivery through information and communication systems. Since the outbreak of the pandemic, 58% of countries are using telemedicine to handle the disruptions in services and reduce in-person consultations.^4^^,^^21^ Accordingly, Saudi Arabia is planning to improve the digital healthcare landscape post-COVID-19, which includes various aspects such as providing care guidance and information, connected medical devices and wearables for monitoring and protection, remote telemedicine, home health robots, using artificial intelligence and machine learning in developing and tracking services, remote monitoring and developing patient networks for increasing healthcare awareness.^22^ Considering these developments, and focusing on the implementation of digital health applications in Saudi Arabia, this article evaluates the Mawid mobile application developed by the Ministry of Health in delivering healthcare services during the COVID-19 pandemic.

## Mawid

**Figure 1.**
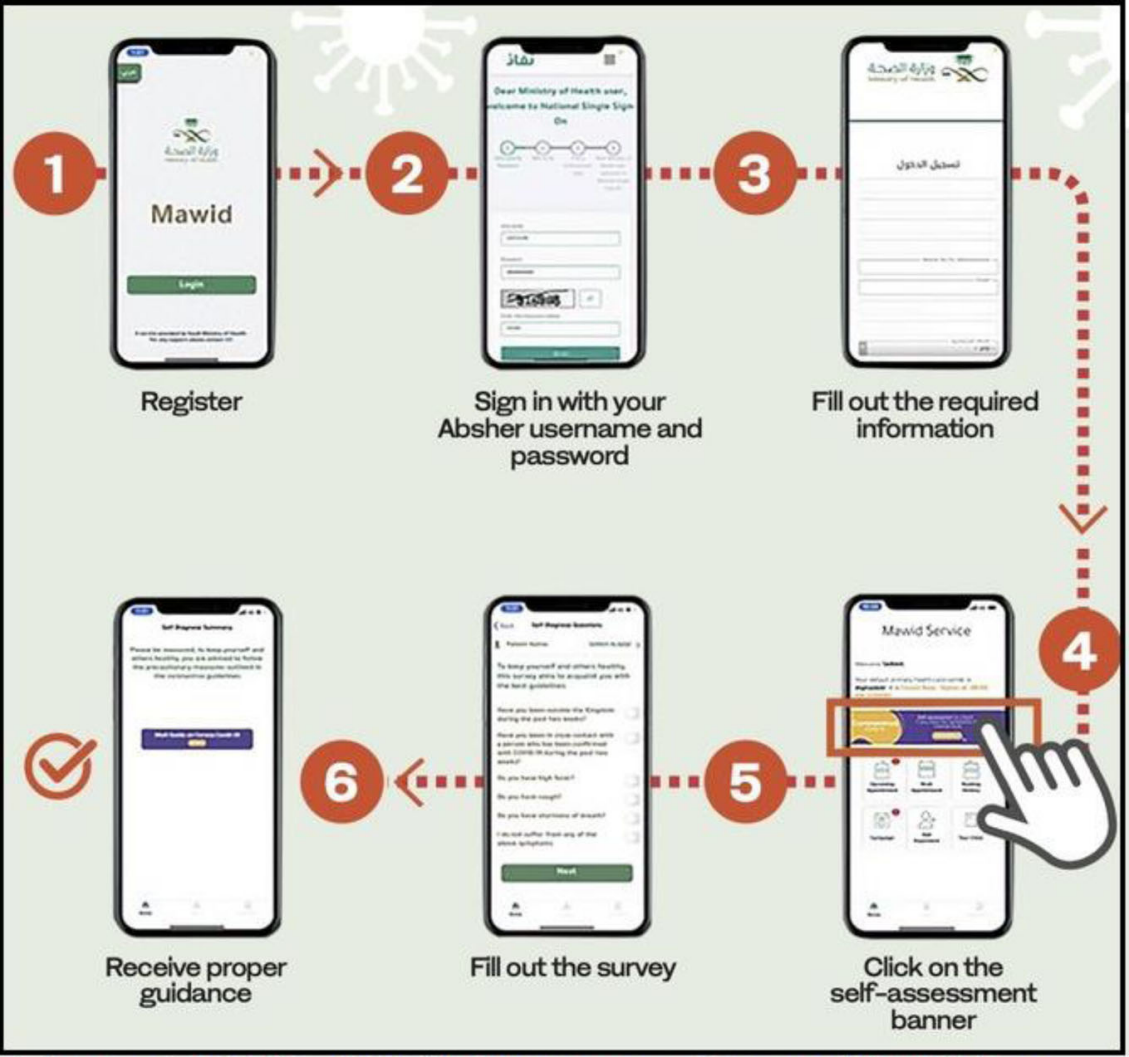
COVID-19 self-assessment test of the Mawid application.

Mawid is a mobile application provided by the Ministry of Health to enable patients to book, cancel and/or reschedule their appointments at primary healthcare centres, as well as manage their referral appointments.^23^ It helps the users to assess the risk of COVID-19 contamination. Users are advised to enter symptoms and their travel details into the application for the risk assessment test. It also helps users in increasing awareness about COVID-19, and the precautionary measures to be taken, in addition to booking, cancelling and/or rescheduling appointments.^24^^,^^25^ The assessment process using the application is presented in Figure 1.

Users can book appointments at 2400 healthcare centres across Saudi Arabia free of cost using the application. In addition, the application has provided >500 000 consultations regarding COVID-19 and >250 000 self-assessment tests have been taken by the users.^25^ Thus the application can be an effective tool for not only delivering healthcare services, but also for tracking and monitoring epidemics. Considering its growing use, the application is evaluated in terms of its usability, user satisfaction, user experience and usefulness.

## Methods

The objective of this study was to evaluate the Mawid mobile healthcare application provided by the Ministry of Health in Saudi Arabia. An online survey was used and the survey questionnaire included questions related to various features of the application. The questionnaire was divided into three sections. The first section included 10 aspects related to ease of use, the second section included 11 aspects related satisfaction and the third section included 11 aspects related to the benefits of using the application. All the factors included in the questionnaire were rated on a scale of 1–5 using a Likert scale^26^ (1, strongly disagree; 2, disagree; 3, neutral; 4, agree; 5, strongly agree). The questionnaire was designed in English (Appendix A), which was later translated into Arabic with the help of a professional translator. The QuestionPro web application was used in preparing the online version of the questionnaire and a survey link was generated for accessing the survey. Fifteen undergraduate students were randomly selected to evaluate the questionnaire in the pilot study. Analysing the results from the pilot study revealed a Cronbach α of 0.81, reflecting reliability and consistency.

## Recruitment

The Mawid application was designed to provide healthcare services to the people of Saudi Arabia. Therefore the sample population in this study included citizens of Saudi Arabia who had accessed the Mawid application at least once. Participants were recruited using the survey link, which was shared on different social media platforms such as WhatsApp, Facebook and Instagram. The survey link was also shared among community groups through e-mails. The survey link was opened on 2 April 2020 and the survey was carried out for 8 weeks, ending on 28 May 2020.

## Sampling

Since the Mawid application can be accessed by any citizen of Saudi Arabia, it was essential that the sample should include people from different regions, in different age groups and of different socio-economic backgrounds. Therefore a random sampling technique was used in selecting the participants. Since the Mawid application can be accessed only by adults, only the adult population was considered for the sample. In addition, a snowball sampling technique was used,^27^ where the participants are requested to forward the survey link to their friends and colleagues using a note in the message attached with the survey link. The survey link was forwarded to 674 people using various social media platforms and community networks. A total of 2542 participants responded to the survey. Of these 2542 participants, 345 incomplete surveys were identified and 204 participants stated that they did not use Mawid application. After removing these 549 invalid responses, a final sample of 1993 was achieved.

## Data analysis

Statistical techniques such as t-tests were used for analysing the data where the results were compared and analysed by age groups and gender. The relationships between different sample groups with respect to the three main factors considered in this study were evaluated by calculating p-values. Descriptive analysis was used for analysing the results, as presented in the next section.

## Results

The participants demographic characteristics are presented in Table 1. The majority of the participants were males and were in the 25–34 y age group. One-fourth of the participants were 18–24 y of age and another one-fourth of participants were 35–44 y of age. Regarding education, 32.2% had a Bachelor's degree, 24.3% had a Master’s degree, 23.9% had a diploma, 2.4% had a PhD and 5.6% completed secondary education. The majority of the participants were private employees, followed by business professionals and public employees. The majority of the participants (83.2%) lived in Riyadh, Mecca and Medina.

**Table 1.** Frequency distribution of demographic variables

Variables	n (%)
Gender	
Male	1186 (59.5)
Female	807 (40.5)
Age (years)	
18–24	553 (27.7)
25–34	771 (38.7)
35–44	425 (21.3)
45–54	229 (11.5)
>54	15 (0.8)
Education	
Secondary	112 (5.6)
Diploma	476 (23.9)
Bachelor's degree	641 (32.2)
Master's degree	485 (24.3)
PhD	48 (2.4)
Other	231 (11.6)
Profession	
Government employee	237 (11.9)
Private-sector employee	824 (41.3)
Business	359 (18.1)
Student	218 (10.9)
Retired	237 (11.9)
Unemployed	118 (5.9)
Region	
Mecca	622 (31.2)
Medina	581 (29.1)
Riyadh	468 (23.5)
Other	322 (16.2)

The participants opinions in relation to the ease of use of the Mawid application reflected high levels of usability and ease of use, as shown in Table 2. Information available on the application, navigation between pages/screens and ease of use were rated as very good by >75% of the participants.

**Table 2.** Ease of use

Questions	Mean	SD
The application was easy to use	4.13	1.5321
It was easy for me to learn to use the app	3.89	1.4365
All users of the application are easy to learn how to use it quickly	3.91	1.1259
I need help while using the application	2.56	1.4358
The information in the app was well organized, so I was easily able to find the information I needed	4.25	1.0097
Taking any action through the application requires a few steps	4.09	1.2174
I used the app without the need for written instructions	3.72	1.3691
The navigation between pages was consistent	4.55	1.2978
Whenever I made a mistake using the app, I could undo it easily and quickly	4.37	1.1315
The app interface allowed me to use all the tasks the app offers, such as entering information, responding to alerts, and viewing information	4.18	1.5416

Further analysing the results by gender, interesting findings were identified. Significant differences between male and female perceptions were identified with respect to ease of use, as shown in Table 3. The mean scores of males (4.21 [standard deviation {SD} 1.51]) and females (3.71 [SD 1.09]) showed that both male and female participants expressed the ease of use of the Mawid application as high. The t-value, as shown in Table 3, was found to be 8.0820 at a 0.05 confidence interval, which was statistically significant (p<0.0001). Therefore significant differences in the ease of use were observed between males and females.

**Table 3.** Difference in ease of use, by gender

Variable	Gender	N	Mean	SD	df	t-Value	p-Value
Difference in ease of use	Male	1186	4.21	1.51	1991	8.0820	<0.0001^*^
	Female	807	3.71	1.09			

* Significant at 0.05 level.

Further analysing the results by age, interesting findings were identified. Significant differences between participants were identified with respect to the ease of use, as shown in Table 4. The mean scores of participants ≤34 y of age (4.28 [SD 1.28]) and participants >34 y of age (3.64 [SD 1.32]) reflected that the participants of all age groups perceived the ease of use to be high. The t-value, as shown in Table 4, was found to be 10.4303 at a 0.05 confidence interval, which is statistically significant (p<0.0001). Therefore significant differences in the experience of ease of use between participants ≤34 y and >34 y of age were observed.

**Table 4.** Difference in ease of use, by age

Variable	Age (years)	N	Mean	SD	df	t-Value	p-Value
Difference in ease of use	≤34	1324	4.28	1.28	1991	10.4303	<0.0001^*^
	>34	669	3.64	1.32			

* Significant at 0.05 level.

The participants’ satisfaction levels with respect to various aspects of the Mawid application are shown in Table 5. Convenience in accessing healthcare services, access to healthcare information, good interface, relevance of tasks and functions on the application were a few aspects with which the participants were highly satisfied, and these factors were rated to be very good by >50% of the participants.

**Table 5.** Users’ satisfaction in using Mawid

Questions	Mean	SD
The app provided a convenient way to access health care services	4.11	1.1522
The application provided sufficient information about the application procedures	4.56	1.5895
I experienced problems while using the application	2.51	1.6124
This app contains all the functions/functions and features I expect	3.68	1.0054
The time I spent using this app was right for me	3.92	1.0548
I liked the application interface	4.55	1.2485
I found all the services, tasks, or functions of the application are well connected	4.28	1.1317
I feel the community is in desperate need of this app	4.41	1.2259
Recommend friends to use this app	4.67	1.5417
I will continue to use the app after the crisis	4.15	1.5698
In general, I am satisfied with this app	4.52	1.2875

Further analysing the results by gender, interesting findings were identified. Significant differences between male and female perceptions were identified with respect to satisfaction levels of using the Mawid application, as shown in Table 6. The mean scores of males (4.25 [SD 1.23]) and females (3.99 [SD 1.37]) reflected that both male and female participants expressed high levels of satisfaction in using the Mawid application. The t-value, as shown in Table 6, was found to be 4.4219 at a 0.05 confidence interval, which was statistically significant (p<0.0001). Therefore significant differences in the satisfaction levels between males and females were observed.

**Table 6.** Difference in satisfaction, by gender

Variable	Gender	N	Mean	SD	df	t-Value	p-Value
Difference in satisfaction	Male	1186	4.25	1.23	1991	4.4219	<0.0001^*^
	Female	807	3.99	1.37			

* Significant at 0.05 level.

Further analysing the results by age, interesting findings were identified. Significant differences between participants were identified with respect to ease of use, as shown in Table 7. The mean scores of participants ≥34 y of age (4.36 [SD 1.15]) and participants >34 y of age (3.89 [SD 1.47]) reflected that the participants of all age groups perceived satisfaction of using the Mawid application to be high. The t-value, as shown in Table 4, was found to be 7.8240 at a 0.05 confidence interval, which was statistically significant (p<0.0001). Therefore, significant differences in the satisfaction levels between participants ≤34 y and >34 y of age can be observed.

**Table 7.** Difference in satisfaction, by age

Variable	Age (years)	N	Mean	SD	df	t-Value	p-Value
Difference in satisfaction	≤34	1324	4.36	1.15	1991	7.8240	<0.0001^*^
	>34	669	3.89	1.47			

* Significant at 0.05 level.

The participants’ perceptions of various benefits of the Mawid application are shown in Table 8. Managing health effectively, delivering effective services remotely, avoiding hospital visits, flexibility in interacting with healthcare providers and ensuring comfort and care by effective delivery of services were a few major benefits identified by participants.

**Table 8.** Benefits of Mawid

Questions	Mean	SD
The application will be beneficial to my health and well-being	4.25	1.5468
The application is more effective in providing the necessary health care compared to the traditional way of going to the hospital personally	4.31	1.2114
Good application from my access to health care services	4.15	1.0812
The app helped me manage my health effectively	3.98	1.0368
A handy app for me communicating with healthcare providers	4.16	1.1547
Using the app, I had more opportunities to communicate/interact with healthcare providers	3.51	1.5874
I felt confident that any information shared with healthcare providers via the app had been received	4.15	1.6315
I felt comfortable communicating with healthcare providers via the app	3.87	1.2457
Using the application saved time	4.54	1.1673
The app fulfilled all my healthcare needs	3.77	1.1874
The application fulfilled all my requirements more easily	3.85	1.5743

## Discussion

Evaluating mobile health applications is important to analyse user acceptance of the application and to identify if the application is able to meet all the users’ needs and expectations.^28^ In addition, it can also help in analysing changes in the users’ needs and attitudes towards the application. Various factors can influence the acceptance of an e-health mobile application. A recent study^29^ identified four factors that can influence users in using the e-health applications: perceived usefulness, perceived ease of use, behavioural intention and risk. Findings related to the ease of use of the application revealed that most of the features of the application were easy to use. The ability to learn, access information and interact with the application were rated as high by the majority of the participants. The perceived risk of the application was identified to be low, as most of the participants considered that the application provided sufficient information about the application procedures. Various benefits perceived by the participants reflect that the application had a behavioural impact, as most of the participants rated higher levels of satisfaction in using the Mawid application. Accordingly, the Mawid application has received high user acceptance levels. In addition, differences between the perceptions of males and females and young and older participants with respect to the ease of use and satisfaction levels were identified, indicating that different genders and age groups have different levels of skills and competencies in using the application and accordingly differ in their attitudes and perceptions towards various features and functionalities of the Mawid application. Therefore there is a need to consider the opinions and needs of both genders and also different age groups in developing a uniform application that fits all.

In relation to the intention to use, the Mawid application was identified as most favourable, as it has benefited its users in various aspects. The findings were similar to those of de Veer et al.,^30^ in which >60% of participants stated that they would use e-health applications. The benefits of the application, such as remote consultation, online treatment and diagnosis, effective communication interface for enhanced communication between patients and doctors, information availability and awareness creation related to COVID-19, have made it one of the most preferred applications in Saudi Arabia. Similar to the Mawid, there are various mobile applications being used in different countries such as India,^31^ China^32^ and Japan.^33^ The Mawid application was shown to be effective in terms of its usability, benefits and user acceptance and satisfaction, thus it is concluded that Mawid application is effective in delivering healthcare services in Saudi Arabia and also effective in tracking and tracing the COVID-19 pandemic. Although the current analysis evaluated the Mawid application as effective, there is a need for regular evaluation and assessment to track changes in users’ needs and update the application according to these changing requirements.

## Limitations

There are a few limitations observed in this study. First was the methodological approach: this study adopted only quantitative methods, using an online survey questionnaire for collecting and analysing the users’ perceptions towards the Mawid application. However, a mixed method approach, adding qualitative approaches such as observations and interviews, could have gathered more qualitative and behavioural data that can be used to further analyse the attitudes and influencing factors related to the Mawid application and its features in light of the COVID-19 outbreak. In addition, the survey was conducted over a period of 8 weeks, which could have been increased to obtain a larger sample population (across the different regions) and higher response rates. Another limitation is that an online questionnaire was used as the instrument for collecting the data, because of the restrictions due to the lockdown.

## Conclusions

Various conclusions can be drawn from the study. First, this study contributes to the literature by providing the perceptions and attitudes of the people towards using e-health applications in light of the COVID-19 outbreak. This information can be used for developing more effective mobile applications for delivering healthcare services and tracking and tracing COVID-19. The findings from the survey proved to be valuable source of information for the government to update its healthcare policies towards using information and communications technology. In addition, issues related to the use of e-health applications such as privacy, miscommunication/misunderstandings on online platforms and risks perceived by the users can be identified and addressed.

## Data Availability

The data that support the findings of this study are available on request from the corresponding author.

## Appendix A: Survey questionnaire

**Evaluation of the Mawid application in delivering services during the COVID-19 p andemic in Saudi Arabia**

The purpose of this survey was to investigate the usefulness, experience, ease of use and satisfaction in using an e-health application (Mawid, developed by the Ministry of Health, Saudi Arabia) for accessing healthcare services by the people in Saudi Arabia during the COVID-19 pandemic. The outcomes from the survey can be used to improve the effectiveness of delivering and managing healthcare services during pandemics through mobile healthcare applications. Kindly participate in the survey and provide your experiences of using the above-mentioned apps.

Contact Person: Dr. Fahad Alanezi

Email: dr_fahed99@hotmail.com

Please click the checkbox to provide your consent for participation in the survey.

**Demographic information**

**Table:**

1. Name
2. Age
18–28
29–50
51–65
3. Gender
Male
Female
4. Education
High school diploma
Some college credits
Associate degree
Bachelor's degree
Master's degree
Professional degree
Doctoral degree
5. Job
Government employee
Private sector employee
Business pioneer
Student
Retired
Others
6. Area of Living
Mecca
Madina El Monawara
Riyadh
Eastern
Qassim
Hail
Tabuk
Northern borders
Joaf
Aseeer
Jazan
Najran
Baha
7. Have you used Mawid application?
Yes
No

**Part A: Ease of use**

Please rate the following items on a scale of 1–5 (1, very poor; 2, poor; 3, neutral; 4, good; 5, very good) based on your experience of using MoH mobile healthcare applications during COVID-19.

1. The application was easy to use
2. It was easy for me to learn to use the app
3. All users of the application are easy to learn how to use it quickly
4. I need help while using the application
5. The information in the app was well organized, so I was easily able to find the information I needed
6. Taking any action through the application requires a few steps
7. I used the app without the need for written instructions
8. The navigation between pages was consistent
9. Whenever I made a mistake using the app, I could undo it easily and quickly
10. The app interface allowed me to use all the tasks the app offers, such as entering information, responding to alerts, and viewing information

**Part B: Satisfaction**

Please rate the following items on a scale of 1–5 (1, extremely unsatisfied; 2, unsatisfied; 3, neutral; 4, satisfied; 5, extremely satisfied) based on your experience of using MoH mobile healthcare applications during COVID-19.

1. The app provided a convenient way to access healthcare services
2. The application provided sufficient information about the application procedures
3. I experienced problems while using the application
4. This app contains all the functions/functions and features I expect
5. The time I spent using this app was right for me
6. I liked the application interface
7. I found all the services, tasks, or functions of the application are well connected
8. I feel the community is in desperate need of this app
9. Recommend friends to use this app
10. I will continue to use the app after the crisis
11. In general, I am satisfied with this app

**Part C: Benefits of Mawid**

Please rate the following items on a scale of 1–5 (1, very poor; 2, poor; 3, neutral; 4, good; 5, very good) based on your experience of using MoH mobile healthcare applications during COVID-19.

1. The application will be beneficial to my health and well-being
2. The application is more effective in providing the necessary healthcare compared to the traditional way of going to the hospital personally
3. Good application from my access to healthcare services
4. The app helped me manage my health effectively
5. A handy app for me communicating with healthcare providers
6. Using the app, I had more opportunities to communicate/interact with healthcare providers
7. I felt confident that any information shared with healthcare providers via the app had been received
8. I felt comfortable communicating with healthcare providers via the app
9. Using the application saved time
10. The app fulfilled all my healthcare needs
11. The application fulfilled all my requirements more easily
